# Use of and Attitudes Toward Technology Among Young People Living With HIV in San Francisco: Cross-Sectional Study

**DOI:** 10.2196/81845

**Published:** 2025-09-15

**Authors:** Sean Arayasirikul, Caitlin Turner, Dillon Trujillo, Jarett Maycott

**Affiliations:** 1 The Legacy Center Joe C Wen School of Population and Public Health University of California, Irvine Irvine, CA United States; 2 Department of Epidemiology and Biostatistics University of California, San Francisco San Francisco, CA United States; 3 Fielding School of Public Health Department of Community Health Sciences University of California, Los Angeles Los Angeles, CA United States; 4 Center for Public Health Research San Francisco Department of Public Health San Francisco, CA United States

**Keywords:** HIV/AIDS, young people living with HIV, digital health, mental health, technology use

## Abstract

Digital technology is an important tool for engaging and delivering care to and for young people living with HIV. This research letter examines how 120 out-of-care young people living with HIV in San Francisco use digital technology, the attitudes they have toward technology, and the anxiety they feel about being without technology. Our findings emphasize the importance of being aware of the unintended consequences of digital health interventions.

## Introduction

The COVID-19 pandemic catalyzed a digital shift in our work and social engagements. Our online world has expanded immensely, with smartphone use increasing from 15% before the pandemic to 36% and tablet/PC use from 2% to 22% [1]. Concerns about this increased screentime and adverse mental health outcomes, especially among young adults, persist despite research in the 1990s and early 2000s [2]. The scientific literature around technology and mental health offers mixed support [2]. Research on the effectiveness of digital and mobile health interventions continues to increase, and the results are promising for many health conditions, especially among young adults with chronic conditions [3]. In this research letter, we present findings on digital technology use among a sample of young people living with HIV and describe their attitudes toward technology and anxiety about being without technology.

## Methods

### Study Design and Participants

We conducted a secondary data analysis of baseline data from a digital HIV navigation intervention study that used text messaging to improve HIV care linkage, retention, and viral suppression among 120 out-of-care young people living with HIV in San Francisco, California. Participants were eligible if they identified as a man who has sex with men or a trans woman, were aged between 18 and 34 years, resided in San Francisco, and were not engaged in HIV care. A digital navigator delivered motivational interviewing, social support, health education, and referrals to participants through text messaging over a period of 6 months. Study procedures were described in a prior manuscript [4].

### Measures and Analysis

We analyzed the following sociodemographic information: age, gender identity, race/ethnicity, education, employment status, and current living situation. To assess use of the internet, mobile phones, and social networking sites [5], participants were asked how often (once a week or less, several times a week, once a day, or several times a day or more) they used email, text messaging, or apps; searched for information; checked social media; or sought out sexual partners or relationships on social networking sites. Positive and negative attitudes toward technology and dependence on technology [5] were assessed using 6, 4, and 4 dichotomous (yes/no) items, respectively. For each of these 3 constructs, items were summed (1/0) and high and low categories created using the median score as a cutoff. To measure psychological distress [6], a composite score was created by summing responses to items ranging from “none of the time” (0) to “all of the time” (5), detailing the frequencies of the following in the last month: feeling calm and peaceful, having a lot of energy, feeling downhearted and blue, and physical health or emotional problems interfering with social activities. Descriptive statistics were used to characterize variables. No missing data were recorded.

### Ethical Considerations

The study protocol was approved by the Institutional Review Board of the University of California, San Francisco (16-19675). Participants provided signed informed consent and were remunerated US $50 for completing the baseline assessment. Data for this secondary analysis were deidentified to protect participants’ privacy and confidentiality.

## Results

The sample was racially and ethnically diverse, with a majority of participants reporting some college or more, unstable housing, and unemployment (56.7%, 67.5%, and 64.2%, respectively). A majority of participants (87.5%) reported using text messaging several times a day or more, followed by mobile phone apps, searching for information on a mobile phone, checking social media, and seeking sexual partners and or relationships on social networking sites (80.8%, 80.0%, 61.7%, and 25.8%, respectively). While most participants (93.3%) reported high levels of positive attitudes toward technology, more than a third (39.2%) reported high levels of negative attitudes toward technology. Many (63.3%) reported high levels of anxiety being without technology. A majority of participants reported being anxious without their cell phone and without internet access (74.2% and 66.7%, respectively). Slightly more than half (53.3%) reported feeling anxious about their personal health information being available online, and nearly two-thirds (65.0%) reported being dependent on technology.

**Table 1:** Demographic characteristics and use of the internet, mobile phones, and social networking sites of a study sample of young people living with HIV in San Francisco, 2016-2019 (N=120; mean age 27.8, SD 4.1 years).

				Participants, n (%)
**Demographics**				
	**Gender identity**			
		Trans woman	17 (14.2)	
		Cisgender man	103 (85.8)	
	**Race/ethnicity**			
		Black, non-Hispanic/Latinx	22 (18.3)	
		Hispanic/Latinx	38 (31.7)	
		Multiple races, non-Hispanic/Latinx	28 (23.3)	
		White, non-Hispanic/Latinx	32 (26.7)	
	**Education**			
		High school/GED^a^ or less	52 (43.3)	
		Some college or more	68 (56.7)	
	**Employment status**			
		Unemployed	77 (64.2)	
		Part-time employed	24 (20.0)	
		Full-time employed	19 (15.8)	
	**Current living situation**			
		Unstable	81 (67.5)	
		Stable	39 (32.5)	
**Use of the internet, mobile phones, and social networking sites**				
	**How often do you send, receive and read e-mails (not including spam or junk mail)? (Choose one)**			
		Once a week or less	24 (20.0)	
		Several times a week	23 (19.2)	
		Once a day	14 (11.7)	
		Several times a day or more	59 (49.2)	
	**How often do you send and receive text messages on a mobile phone?**			
		Once a week or less	8 (6.7)	
		Several times a week	3 (2.5)	
		Once a day	4 (3.3)	
		Several times a day or more	105 (87.5)	
	**How often do you use apps (for any purpose) on a mobile phone?**			
		Once a week or less	12 (10.0)	
		Several times a week	6 (5.0)	
		Once a day	5 (4.2)	
		Several times a day or more	97 (80.8)	
	**How often do you search for information with a mobile phone?**			
		Once a week or less	9 (7.5)	
		Several times a week	10 (8.3)	
		Once a day	5 (4.2)	
		Several times a day or more	96 (80.0)	
	**How often do you check your social media page from your smartphone?**			
		Once a week or less	27 (22.5)	
		Several times a week	12 (10.0)	
		Once a day	7 (5.8)	
		Several times a day or more	74 (61.7)	
	**How often do you seek sexual partners and or relationships on social networking sites?**			
		Once a week or less	72 (60.0)	
		Several times a week	16 (13.3)	
		Once a day	1 (0.8)	
		Several times a day or more	31 (25.8)	

^a^General Educational Development.

**Table 2:** Attitudes toward technology and anxiety about being without technology among young people living with HIV in San Francisco, 2016-2019 (N=120).

			Participants, n (%)
**Positive attitudes toward technology**			
	**I feel it is important to be able to find any information whenever I want online.**		
		No	15 (12.5)
		Yes	105 (87.5)
	**I feel it is important to be able to access my personal health information whenever I want online.**		
		No	16 (13.3)
		Yes	104 (86.7)
	**I feel it is important to be able to access the Internet any time I want.**		
		No	12 (10.0)
		Yes	108 (90.0)
	**I think it is important to keep up with the latest trends in technology.**		
		No	29 (24.2)
		Yes	91 (75.8)
	**Technology will provide solutions to many of our problems.**		
		No	34 (28.3)
		Yes	86 (71.7)
	**With technology anything is possible.**		
		No	34 (28.3)
		Yes	86 (71.7)
	**I feel that I get more accomplished because of technology.**		
		No	31 (25.8)
		Yes	89 (74.2)
	**Mean composite score (mean score 5.6, SD 1.8)**		
		Low	8 (6.7)
		High	112 (93.3)
**Negative attitudes toward technology**			
	**New technology makes people waste too much time.**		
		No	68 (56.7)
		Yes	52 (43.3)
	**New technology makes life more complicated.**		
		No	80 (66.7)
		Yes	40 (33.3)
	**New technology makes people more isolated.**		
		No	56 (46.7)
		Yes	64 (53.3)
	**Mean composite score (mean score 1.3, SD 1.2)**		
		Low	73 (60.8)
		High	47 (39.2)
**Anxiety about being without technology**			
	**I get anxious when I don’t have my cell phone.**		
		No	31 (25.8)
		Yes	89 (74.2)
	**I get anxious when I don't have the Internet available to me.**		
		No	40 (33.3)
		Yes	80 (66.7)
	**I am anxious about having my personal health information available online.**		
		No	64 (53.3)
		Yes	56 (46.7)
	**I am dependent on my technology.**		
		No	42 (35.0)
		Yes	78 (65.0)
	**Mean composite score (mean score 2.5, SD 1.5)**		
		Low	44 (36.7)
		High	76 (63.3)

## Discussion

We found that many young people living with HIV used mobile phones, social media, apps, and social networking sites on a regular basis. This high use may be a factor in participants’ experience of anxiety being without these digital technologies. Other research has found that technology dependence is an important consideration to shape the care of young people [7]. Furthermore, technology can exacerbate isolation, negative social comparison, loneliness, and other poor mental health outcomes [8]. This study has limited generalizability due to its study design and its focus on HIV care engagement among young people living with HIV. Despite this, it is critical to consider not only how digital technology can improve health, but also its influence on technology dependence and anxiety as unintended consequences [9].

## Data Availability

The datasets generated or analyzed during this study are not publicly available as they contain details that could be used to identify participants but are available from the corresponding author on reasonable request.

## References

[1] Werling AM, Walitza S, Drechsler R (2021). Impact of the COVID-19 lockdown on screen media use in patients referred for ADHD to child and adolescent psychiatry: an introduction to problematic use of the internet in ADHD and results of a survey. J Neural Transm (Vienna).

[2] George MJ, Russell MA, Piontak JR, Odgers CL (2018). Concurrent and subsequent associations between daily digital technology use and high-risk adolescents' mental health symptoms. Child Dev.

[3] Rathbone AL, Prescott J (2017). The use of mobile apps and SMS messaging as physical and mental health interventions: systematic review. J Med Internet Res.

[4] Arayasirikul S, Trujillo D, Turner CM, Le V, Wilson EC (2019). Implementing a digital HIV care navigation intervention (Health eNav): protocol for a feasibility study. JMIR Res Protoc.

[5] Rosen LD, Whaling K, Carrier LM, Cheever NA, Rokkum J (2013). The Media and Technology Usage and Attitudes Scale: An empirical investigation. Comput Human Behav.

[6] Ware J, Kosinski M, Keller SD (1996). A 12-Item Short-Form Health Survey: construction of scales and preliminary tests of reliability and validity. Med Care.

[7] Toly VB, Blanchette JE, Al-Shammari T, Musil CM (2019). Caring for technology-dependent children at home: problems and solutions identified by mothers. Appl Nurs Res.

[8] Ponnusamy S, Iranmanesh M, Foroughi B, Hyun S (2020). Drivers and outcomes of Instagram addiction: psychological well-being as moderator. Comput Human Behav.

[9] Cao W, Cao X, Sutherland AD (2025). Planning for the unexpected and unintended effects of mHealth interventions: systematic review. J Med Internet Res.

